# 

**Published:** 1896-10

**Authors:** 


					﻿Vol. XVI.
OCTOBER, 1896.
NO. 10;
THE
pOMEOPATHlC pHY^lClAfl,
A Monthly Journal of Homoeopathic Materia Medica
and Clinical Medicine.
•“ He is a freeman whom truth makes free, and all are slaves beside."
"Seek the Truth: come whence it may, cost what it will."
EDITED AND PUBLISHED BT
WALTER Næ. JAMES, M. D.
PHILADELPHIA:
ITo. 1231 LOCUST STREET.
londo n :■
ALFRED HEATH & CO., Sole Agents fob Great Britain,:
114 Ebury Street, Eaton Square.
Yearly Subscription, $2.50, invariably in advance. Single numbers, 25 els.
Entered at the Philadelphia Post-OSoe as Second-Class Mall Matter.
				

